# Female Asthma Has a Negative Effect on Fertility: What Is the Connection?

**DOI:** 10.1155/2014/131092

**Published:** 2014-03-27

**Authors:** Elisabeth Juul Gade, Simon Francis Thomsen, Svend Lindenberg, Vibeke Backer

**Affiliations:** ^1^Respiratory Research Unit, Department of Respiratory Medicine, Bispebjerg University Hospital, Bispebjerg Bakke 23, 2400 Copenhagen NV, Denmark; ^2^Copenhagen Fertility Center, Infertility Clinic, Lygten 2C, 2400 Copenhagen NV, Denmark; ^3^Department of Dermatology, Bispebjerg University Hospital, Bispebjerg Bakke 23, 2400 Copenhagen NV, Denmark

## Abstract

Reproductive changes such as impaired fertility and adverse pregnancy outcomes have been related to female asthma. We recently found that time to pregnancy is prolonged in asthmatic females especially in women with moderate to severe asthma and in those above 30 years of age. Despite their reproductive difficulties the asthmatics ultimately conceived just as many biological children as healthy throughout their reproductive lives. This knowledge therefore raises questions about how asthma affects fertility pathophysiologically. The purpose of this review is to describe the existing knowledge in this field and suggest hypotheses of causal relationships, which may form the basis for future studies in this field. The aim is, in particular, in the literature to examine whether there is any evidence to suggest that the systemic inflammation that characterizes asthma, can affect fertility. The issue is potentially clinically important for asthmatic, infertile individuals and society because treatment of the general systemic inflammation associated with the asthmatic disease combined with hormone stimulation might be the optimal target for an effective infertility therapy, possibly decreasing the need for in vitro fertilization.

## 1. Introduction

We have recently, in a retrospective registry-based study, demonstrated that asthma significantly prolongs time to pregnancy (TTP) and thus fertility (21,6% versus 27% OR: 1.31 *P* = 0.009), whereas allergy was not related to TTP [1]. Furthermore we showed that untreated asthmatics had a significantly increased risk of prolonged TTP compared with healthy individuals (30.5 versus 21.6%, OR = 1.79, *P* = 0.004), while asthmatics receiving any kind of treatment for asthma tended to have a shorter TTP than untreated asthmatics (23.8 versus 30.5%, OR = 1.40, *P* = 0.134) indicating that an untreated systemic inflammation could have a negative effect on fertility. These findings are to our knowledge new and of highly clinical relevance given that a large proportion of asthmatic patients are young and therefore in the reproductive age.

The reasons for our findings are, however, unknown. The purpose of this review is therefore to provide an overview of the basic literature of the pathophysiology behind this finding and to suggest hypotheses on causal relationships.

So far only few other studies have examined this issue, and even so, the results have been conflicting as to whether asthma or other atopic diseases can affect fertility. Other recent studies by Carson et al. [2] and Harju et al. [3] suggest an association between subfertility, fertility treatment, and asthma in children born through in vitro fertilization (IVF), whereas another study showed no increased risk of asthma or other atopic diseases in children born after IVF [4]. An association could be due to both the IVF treatment itself or a genetic predisposition to asthma. Furthermore Källén and Otterblad Olausson [5] suggest that a greater proportion of asthmatic women seek fertility treatment, indicating a link between asthma and infertility. They reported that a greater number of women undergoing IVF treatment were using antiasthmatic drugs.

In contrast to the above, Tata et al. [6] found no evidence supporting that fertility rates (live births pr. 1000 person years) of women with asthma, eczema, or hay fever were lower than healthy women. Contrary to this, Tata et al. found that women with hay fever or eczema were more fertile than those without these conditions. Furthermore, asthma was hypothesized to have a different relationship to fertility than eczema and hay fever, as young asthmatic individuals had a higher fertility rate than their older counterparts compared with the healthy group. This is in agreement with other findings [1].

Although it seems difficult to draw a conclusion based on the few available studies, an association between asthma and infertility seems plausible, since an interaction between the severity of asthma and other aspects of the female reproductive health, with respect to the menstrual cycle, abortions, and pregnancy, has already been shown—though poorly understood [7–9].

## 2. Method

Searches were conducted on MEDLINE, Embase, and Cochrane Library together with reference lists of retrieved papers. Papers from 2010 to 2013 provided the basis for this literature review, but a few considerably older articles from 1989 onwards were also used due to the importance of the material and because the knowledge had not been expanded yet.

Keywords used were asthma AND systemic inflammation, inflammation AND infertility, endometrial respectability, asthma AND fertility, Polycystic Ovarian Syndrome (PCO) AND asthma, endometriosis AND asthma, metabolic syndrome AND asthma.

### 2.1. Asthma and Inflammation

The mechanisms linking asthma to fertility still remain poorly investigated. However, recent data [1] indicate that the nature and degree of inflammation characterizing asthma are important since nonatopic asthma, untreated asthma, and moderate to severe asthma had the largest effect on fertility, that is, increased TTP. We therefore propose that the asthmatic inflammation is a universal problem compared to allergy alone and is involving mucosal surfaces other than the bronchi. Accordingly, one assumption is that the same inflammation and increased number of inflammatory cells and mediators are also found in the uterus or the fallopian tubes in asthmatic women.

Furthermore, we propose that the impact asthma has on fertility is dependent on the asthmatic phenotype and the particular kind of inflammation that characterizes each phenotype. This may prove important in future possible treatment attempts to reduce TTP in asthmatics.

In recent years, our understanding of the pathogenesis of asthma has changed dramatically. It is now clear that T helper cells other than Th2 cells are involved in the pathogenesis of asthma; specifically, both Th1 and Th17 cells are crucial for the development of neutrophilic inflammation in the airways. The disease is therefore believed to be heterogeneous, which depending on its phenotype varies both in terms of clinical expression, the degree and nature of inflammation, and response to treatment. There is no clear consensus on how the phenotypes should be divided, but it is recognized that asthma can be classified as eosinophilic asthma (EA) and noneosinophilic asthma (NEA) based on the presence of eosinophils in sputum. EA is often seen in atopics where allergen exposure triggers inflammation and response. It may also occur in nonatopics where other factors of nonallergic origin are of importance. EA is characterized by a disturbed balance between Th1 and Th2 cytokines—with dominance towards Th2 cytokines. The eosinophilic inflammation is generated when antigen specific-IgE binds to mast cells and basophils in the airway to course a local allergic response. IL-33 and IL-25 produced by epithelial cells then promote IL-13 and IL-5 release from IL-25 receptor natural helper cells in the airway. This cascade promotes Th2 cell differentiation and further production of the Th2 cytokines IL-4, IL-5, and IL-13 locally. Furthermore, T and B cells are generated to facilitate more rapid responses to repeated stimulation and disease chronicity. Consequences of IL-13 exposure on lung cells in asthma include goblet cell metaplasia, increased mucous production, airway fibrosis, airway smooth muscle hyperresponsiveness, and remodeling.

A large number of asthmatics, especially the elderly, have an underlying pathology that is different from Th2 eosinophilic asthma. This phenotype—NEA—is characterised by higher numbers of serum and sputum neutrophils [10]. Many of these patients have severe, persistent asthma in the absence of eosinophilic inflammation and are unresponsive to corticosteroids, showing more extensive structural changes [11]. There are markedly different patterns of inflammatory cells and mediators involved [12], since this type is associated with a more Th1-driven response [11]. The cascade of inflammation is initiated by a neutrophil influx and activation, which may be mediated by IL-8 secretion [13]. This neutrophilic activation increases Th17 cytokines (IL-17A, IL-17F, and IL-22) and is recruiting neutrophils to the airway by increasing secretion of epithelial-derived neutrophilic chemokines. In addition, Th17 cytokines also induce mucous cell metaplasia and have pleotropic effects on airway smooth muscle resulting in airway narrowing [14].

To date, most data support a systemic pathway linking the upper and lower airways in asthma, involving both the bloodstream, bone marrow, and thereby the whole body [15, 16]. This indicates that asthma affects not only the organ of origin but also other systems in different parts of the body, probably mediated by the bloodstream. The exact nature of these relationships is not fully understood, but there is increasing evidence to support bone marrow stimulation resulting in an increase in blood progenitors for eosinophil/basophils and IL5 levels. These cells migrate not only to the involved mucosa, but also to other target organs [17, 18]. The present data support the notion that asthma causing chronic peripheral inflammation modifies the whole body's inflammatory status and thereby also the reproductive organs.

The systemic inflammation in asthma is not fully understood, but data indicate that it can be traced by detection of elevated proinflammatory cytokines in the peripheral blood in the form of IL-6, tumor necrosis factor-*α* (TNF-*α*), that stimulate hepatic production of CRP, and increased neutrophils and natural killer cells. In particular, plasma CRP has been shown to be elevated in nonallergic and steroid-naive patients and is correlated with increased inflammatory cell count [19]. The systemic inflammation is therefore assumed to be more pronounced in the neutrophilic phenotype, which agrees well with our hypothesis and our findings that noneosinophilic asthmatics have the longest TTP [1].

To date, guidelines for asthma treatment have not changed to favor systemic therapy or phenotype, but the understanding is that this is necessary to progress from relieving to treating the disease and thereby also its comorbidities. Currently available systemic therapies are, for example, leukotriene receptor antagonists, as well as biological agents such as anti-IgE, anti-IL-17, and anti-IL5, which may have more targeted immune modulatory properties. Treatment of asthma as a systemic disease requires clinical trials that evaluate the effects of new treatments on both lung function and the wider systemic pathology [20].

### 2.2. Asthmas Association with Gynaecological Diseases

There are several studies that indicate both coherence between asthma and endometriosis as well as a correlation between metabolic syndrome/PCOS and asthma.

Studies of women with endometriosis show a higher prevalence of asthma than among the general population [21, 22]. A common inflammatory pathway has been indicated as an explanation, as leukotriene antagonists intended for asthma treatment have been found useful in the treatment of endometriosis [23].

Furthermore, there is growing evidence that the metabolic syndrome (abdominal obesity, insulin resistance, hypertension, and dyslipidaemia) is a strong risk factor for asthma, even stronger than obesity [24]. A study by van Huisstede et al. [25] showed an association between eosinophilia, lung function, and metabolic syndrome, while BMI had no correlation to this.

Others have correlated PCOS to asthma and lower lung function and concluded that airways pathology might have not only a hormonal but also a metabolic component [26].

Common features of PCOS, metabolic syndrome, and endometriosis are subfertility, systemic inflammation, and increased number of spontaneous abortions [27–29] and an association to asthma could therefore, to some extent, explain the reduced fertility in asthmatics.

These data substantiate the hypothesis of an association between asthma and infertility.

### 2.3. Systemic Inflammation and Fertility

A key issue is how a shift in the body's usual inflammatory level can affect fertility.

Cytokines and growth factors are suggested to play an important role in the initial process of successful implantation and are also deeply involved in the inflammatory response that characterizes asthma. For many years Th2 immunity was assumed supportive of normal pregnancy by preventing rejection of the “foreign” fetal tissue, while Th1 immunity was considered detrimental to the fetus. In particular, the balance between them was considered important [30].

In recent years, the belief regarding the immune system's involvement in fertility has changed. An imbalance of the adaptive immune system (both the Th1 and Th2/TH17 response) is now associated with infertility [31]. The assumption is that a small amount of inflammation is conducive to pregnancy, while too much is destructive, regardless of which response is initiated.

Following this hypothesis, it seems likely that the asthmatic disease can affect fertility by causing an imbalance in the adaptive immune system towards either Th1/Th17 or Th2.

In particular, TNF*α*'s role in conception and pregnancy supports the assumption of the dual role of the Th1 response. TNF*α* is part of the Th1 cell response, which is assumed to play an important part in reproductive failure but is at the same time essential for trophoblastic cell growth, cell differentiation, and angiogenesis, thereby promoting implantation.

Special focus has been on IL6, a key mediator of immune and acute phase responses, as several studies indicate that dysregulation of IL-6 may disturb implantation and be harmful to pregnancy [32, 33]. IL-6 is a key cytokine that blocks the development of regulatory T (Treg) cells and induces the differentiation of Th17 cells. Th17 induces the expression of many inflammatory mediators. Recent data show an increased prevalence of Th17 cells in peripheral blood and decidua among patients with unexplained recurrent spontaneous abortions [34]. The consequence for fertility of an imbalanced IL6 is unknown, but data suggest that IL6-type cytokines play an important role during implantation and are able to regulate the survival, proliferation, and differentiation of fetal murine germ cells. Evidence from infertile women supports the notion that aberrant expression of cytokine IL6, or the related LIF in the endometrium, is associated with recurrent miscarriages and failed implantation. Some suggest that this is due to a higher cytotoxic NK count [35–37]. These findings are interesting, as IL6 is produced in the lungs of asthmatic patients [38]. Asthmatic women are therefore presumed to have a higher concentration of circulating IL6 than healthy, nonasthmatic individuals, which could impair their fertility.

Although the systemic effects of IL6 and its origin are already characterised, it is only partially understood because of its complexity in the body, as it has both an immunological and metabolic origin and effect. The immunological effect is most likely dual: at some levels it acts as a defence mechanism, but in chronic inflammation and disease it is proinflammatory rather than a defence such as in rheumatoid arthritis and inflammatory bowel disease.

In the metabolic system, IL6 originates from adipose tissue and muscle cells, and its effect is controversial. Some believe that IL6 has a dual effect; if generated from adipose tissue, it may cause insulin resistance and type 2 diabetes, and if formed from muscle cells during hard physical activity, it results in increased insulin sensitivity [39]. Others believe that IL6 is the cross-link and connection between insulin sensitive tissue and insulin production and is thereby “the good team player” [40]. Many questions concerning IL6 action and its interaction between the metabolic and the immunological function are so far unanswered. The immunological effects of IL6 have led to inhibition of the IL6 pathway being proposed as an effective prevention and treatment of inflammatory diseases such as asthma [41]. Studies show that blocking the IL6 receptor leads to downregulation of the Th2 cytokines IL4, IL5, and IL13 and thereby downregulation of the eosinophilic asthmatic response [42, 43]. This might be a target for infertile eosinophilic asthmatics in the future.

### 2.4. The Uterus and Systemic Inflammation

The general belief is that some degree of systemic or uterine inflammation is essential for normal function of the ovary, uterus, embryo, implantation, and pregnancy. Several studies show that an endometrial biopsy, which induces an inflammatory response, facilitates the preparation of the endometrium for implantation and has a positive correlation with pregnancy outcome [44]. However, when inflammation becomes too excessive, chronic, or consists of specific mediators in high concentration (IL6), it might cause degeneration of the embryo and impaired implantation [45]. It has been indicated that subclinical endometrial inflammation could play a role in implantation failure after IVF because inflammatory mediators, such as cytokines and chemokines, can cause trophoblastic apoptosis and the cascade of events leading to implantation failure [46]. Other inflammatory mediators common to the reproductive organs and asthma are natural killer cells. Peripheral natural killer (pNK) and uterine NK (uNK) cells have been associated with reproductive failure. The NK cells in general regulate trophoblastic invasion by production of IL8 and IL10 chemokines in human decidua. Peripheral blood NK activity is shown to be significantly higher in women with repeated IVF failures than among fertile controls. Presumably this is because pNK cells use their cytotoxic functions, while uNKs mediate pregnancy by promoting angiogenesis and trophoblastic chemoattraction. In regard to asthma, studies have indicated that NK cells are enhanced in peripheral blood and involved in both the promotion and inhibition of lung inflammation [47]. The prognostic value of measuring pNK or uNK cell parameters remains uncertain [42, 48, 49].

Boomsma et al. [50] proposed that glucocorticoids could improve fertilization by acting as immune modulators to reduce the uterine NK cell count and normalize the cytokine expression profile in the endometrium by suppressing endometrial inflammation. They showed that use of glucocorticoids in women undergoing IVF was associated with a borderline statistically significant improvement in pregnancy rates.

The important issue when discussing a significant coexistence between asthma and fertility is whether a systemic disease not connected to the reproductive organs can affect the uterine mucosa and thereby fertility. There are only few data on this topic. Some studies have by biopsy shown that systemic diseases such as sarcoidosis or tuberculosis can affect the endometrial mucosa by causing granulomatous inflammation in the endometrial lining [51]. Another study by Kurganov [52] in rodents showed that the endometrial mucosa during and after a systemic infection/chronic inflammation was infiltrated by eosinophils and that the infiltrated endometrium was unable to incorporate the embryonated egg. Hence it is possible that other systemic (inflammatory) diseases can affect the endometrial lining too.

## 3. Conclusion

Based on the available evidence, we know that asthma and maybe other systemic inflammatory diseases can have negative influence on fertility. The association increases with the degree and nature of the inflammation, and data suggest that the severity of asthma and the treatment level as well as the asthma phenotype can increase this tendency. Our assumption is that asthma in the lower airways can simultaneously cause inflammation in the mucosa in the uterus due to a systemic reaction, and that this inflammation, if not treated correctly, can inhibit normal implantation of the fertilized ovum. This assumption seems plausible because many of the same inflammatory cytokines that are destructive to reproductive health are also present systemically in atopic individuals.

Our assumption is strengthened by the fact that sarcoidosis, a well-known inflammatory lung disease affecting the whole organism, can change the composition of cells in the lining of the uterus and provoke inflammation without causing any symptoms from the reproductive organs.

Furthermore, it seems evident that female asthmatics may have fertility problems, since there seems to be a link between asthma and increased TTP as well as asthma and several gynaecological diseases, with a negative effect on fertility.

To clarify this issue research is needed with a larger number of female asthmatic, infertile patients aimed at clarifying the endometrial changes caused by systemic inflammatory diseases.

If there is a connection between systemic inflammation and infertility, the IL6 pathway or pathways for other cytokines (NK cells) could be an experimental target for treatment, possibly forming the basis for a successful treatment for all patients with inflammatory diseases and fertility problems. Far more knowledge is needed about the effect of the immune system as well as the effect of asthma on fertility before there is hope for a possible treatment.
